# Corticomuscular Coherence Analysis on Hand Movement Distinction for Active Rehabilitation

**DOI:** 10.1155/2013/908591

**Published:** 2013-04-16

**Authors:** Xinxin Lou, Siyuan Xiao, Yu Qi, Xiaoling Hu, Yiwen Wang, Xiaoxiang Zheng

**Affiliations:** ^1^Qiushi Academy for Advanced Studies, Zhejiang University, Hangzhou, Zhejiang 310027, China; ^2^College of Biomedical Engineering & Instrument Science, Zhejiang University, Hangzhou, Zhejiang 310027, China; ^3^School of Computer Science and Technology, Zhejiang University, Hangzhou, Zhejiang 310027, China; ^4^Interdisciplinary Division of Biomedical Engineering, Hong Kong Polytechnic University, Hong Kong; ^5^Key Laboratory of Biomedical Engineering of Ministry of Education, Zhejiang University, Hangzhou, Zhejiang 310027, China

## Abstract

Active rehabilitation involves patient's voluntary thoughts as the control signals of restore device to assist stroke rehabilitation. Although restoration of hand opening stands importantly in patient's daily life, it is difficult to distinguish the voluntary finger extension from thumb adduction and finger flexion using stroke patients' electroencephalography (EMG) on single muscle activity. We propose to implement corticomuscular coherence analysis on electroencephalography (EEG) and EMG signals on Extensor Digitorum to extract their intention involved in hand opening. EEG and EMG signals of 8 subjects are simultaneously collected when executing 4 hand movement tasks (finger extension, thumb adduction, finger flexion, and rest). We explore the spatial and temporal distribution of the coherence and observe statistically significant corticomuscular coherence appearing at left motor cortical area and different patterns within beta frequency range for 4 movement tasks. Linear discriminate analysis is applied on the coherence pattern to distinguish finger extension from thumb adduction, finger flexion, and rest. The classification results are greater than those by EEG only. The results indicate the possibility to detect voluntary hand opening based on coherence analysis between single muscle EMG signal and single EEG channel located in motor cortical area, which potentially helps active hand rehabilitation for stroke patients.

## 1. Introduction

Stroke is one of the leading causes of death in the world [1, 2]. In addition to the high death rate, most stroke patients may lose many daily activities, such as walking, grasping, and speaking [3]. To restore the losing motor functions of a stroke patient, rehabilitation therapies are often necessary and proven to be effective [4–9]. Studies on fMRI, PET, and TMS had shown that some areas of the stroke patient's brain indicated reorganization [8–10], which played an important role for restore of patients' function. There are mainly two kinds of rehabilitation: passive rehabilitation and active rehabilitation. The passive rehabilitation directly stimulates the affected muscles in therapies without involving patients' volition, such as physical training, electrical stimulation (ES) [11–14]. On the contrary, active rehabilitation is that stroke patients' volition is a necessary part in rehabilitation, in which the muscles are stimulated to be active only when the patients intent to do so. More importantly, active rehabilitation was proved to be more effective to restore stroke patient' motor function and improve the performance of brain plasticity [15–17]. Electromyography (EMG) or electroencephalography (EEG) signals had been utilized and proved to be as useful tools when volition was involved in active rehabilitation [18–21]. EMG, as the control signals of ES, could be adopted to help restore stroke patients' walk and grasp functions [18–20]. However, the muscles are usually coactive, spastic, or amyotrophic for stroke patients [22]. The voluntary intention extract from such signals may not be reliable.

In therapies, restoration of hand function is usually a common procedure for stroke patients, since the loss of normal hand function can cause great difficulties in daily life. One of the most challenge movements is that the therapist needs to help patient to open hand, because the patient's hand is usually spastically closed [23, 24]. Extensor Digitorum is the muscle that mainly involved in finger extension (corresponding to hand opening). Although finger extension can be differentiated from thumb adduction and finger flexion (corresponding to hand closing) with EMG signals on extensor digitorum [25–27] for healthy subjects, it is not easy to classify different hand movements for stroke patients, since the muscles are coactive, spastic, or amyotrophic [22]. Therefore, extracting the intention of single muscle activity that involved in hand opening, and distinguishing from the hand closing, is meaningful for hand function restoration. 

EEG is one of the approaches to interpret patient movement intentions [21]. For example, motor imagery (MI) can be used to distinguish rest from movements, or right from left motor imagery. In Pfurtscheller and Neuper's study, ES was controlled by EEG signals to help patient with spinal cord injury to restore grasp function [21]. However, EEG in his study utilize MI that could only distinguish left from right movement and it is difficult to classify different ipsilateral hand actions, such as finger extension (corresponding to hand opening), thumb adduction, and finger flexion (corresponding to hand closing) [28–31]. Since EMG signals are induced from EEG signals, the coherence is observed only in correlated EEG-EMG signals [32]. Even though coactive muscles or spasticity was observed in stroke patient's ipsilateral hand muscle, the cortico-muscular coherence (CMC) may not exist, because the collected EMG signals do not involve patient volition [32]. Conway et al. firstly described CMC existing in magnetocephalography (MEG) and first dorsal interosseous muscles surface EMG [33]. Mima and Hallett extracted coherence between EEG and right Abductor Pollicis Brevis muscle EMG and described CMC mechanism [34, 35]. The arm and hand coherence had overlap area shown in Mima and Steger's study [36].

In this paper, we propose to analyze cortico-muscular coherence between EMG and EEG signals to distinguish voluntary ipsilateral hand opening, hand closing, and rest state. The EMG signals of Extensor Digitorum (ED) muscle and 35 channels of EEG signals are simultaneously collected when 8 subjects are instructed to voluntarily execute right finger extension, thumb adduction, finger flexion, and rest. We observe the spatial distribution of the EEG channels when the cortico-muscular coherence reaches peak value. Then brain channel corresponding to the voluntary movement is fixed in left motor cortex, and CMC value over different frequency within beta range is explored for 4 different executions. After the temporal and spatial feature extraction, we apply *t*-test to check if the coherence between EMG on ED and EEG signals is statistically different among 4 movement states across all subjects. We finally implement linear discriminate analysis to classify finger extension from thumb adduction, from finger flexion, and from rest states based on cortico-muscular coherence value. 

## 2. Materials and Methods

### 2.1. Subjects and Experiments Paradigm

We recruit 8 normal right-handed volunteers in this study without any healthy neurology disease history (7 males and 1 female, mean age 24.13 ± 1.36, as shown in Table 1). The subject's handedness is tested by the Edinburgh inventory [37]. All subjects are given informed written consent in the experiment and the protocol is approved by the ethics committees Zhejiang University. 

**Figure 1:**
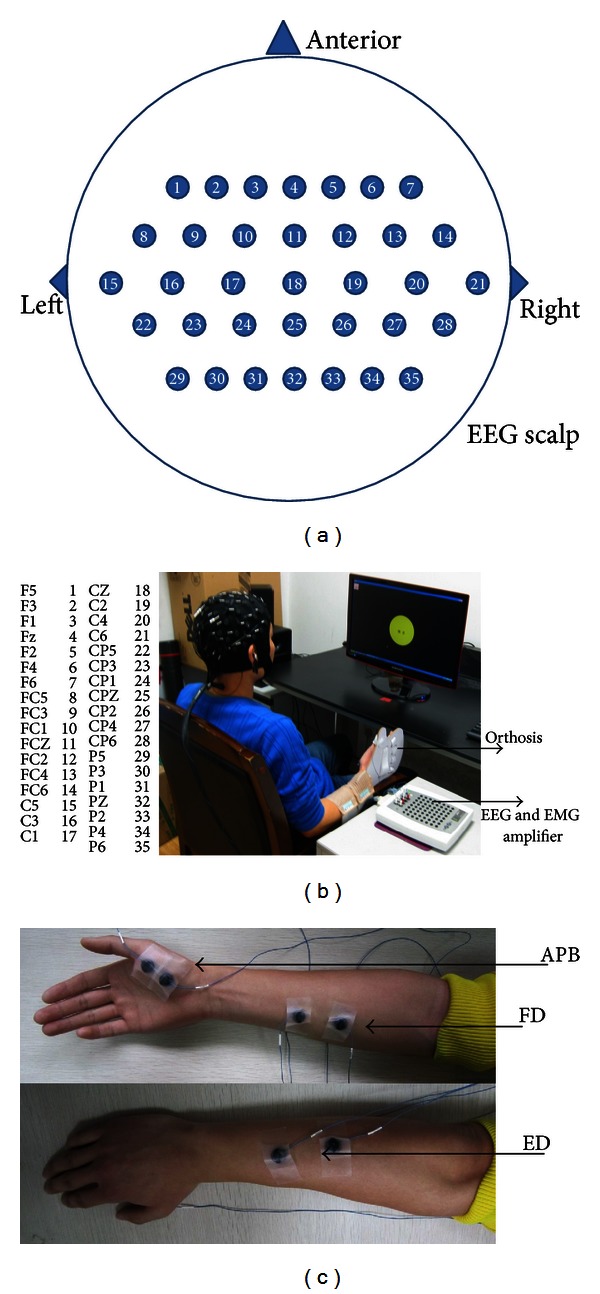
(a) Top view of 35 channel quick cap and the mapping to 64 Ch quick cap name. (b) Demonstration of the experiment setup. (c) Extensor Digitorum (ED), Abductor Pollicis Brevis (APB), and Flexor Digitorum (FD) muscles position.

The subjects are seated comfortably in front of table and asked to perform four simple hand movement tasks (finger extension (corresponding to hand opening), thumb adduction, finger flexion (corresponding to hand closing) and rest). The first three hand movements mainly correspond to Extensor Digitorum (ED), Abductor Pollicis Brevis (APB) and Flexor Digitorum (FD) muscles, respectively. In order to standardize experiment condition, an orthosis is used to fix subjects' finger and upper limb is fixed on the armrest (as shown in Figure 1(b)).

The total experiment contains two main parts. Firstly, the subjects are asked to finish the maximum volunteer contraction (MVC) test [38]. To compare different subject and same subject at different time, MVC test is necessary, because EMG amplitudes are different among subjects, so are the maximum EMG of the same subject. The subjects are asked to do three separately above-mentioned muscle MVC test, and the hand is fixed by a splint (as shown in Figure 1(b)). We take ED muscle for an example here. 

**Figure 2:**
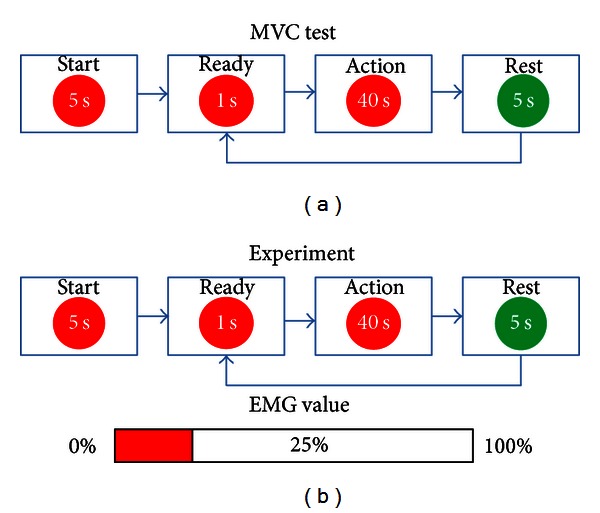
(a) The scheme of the MVC test and (b) upper is the scheme of the experiment and bottom is the real feedback bar. The subject is asked to maintain 25%  EMG_max⁡_.

There are three states in whole MVC test hinted on monitor (as shown in Figure 2(a)), ready, action, and rest. The subject should keep 10 s maximum finger extension with action hint in the monitor. The subject is asked to finish MVC test three times for each muscle. The average of this duration EMG amplitude is donated as EMG_max⁡_ [38].

After the MVC test, each subject is asked to activate the target muscle with amplitude close to 25% of EMG_max⁡_ as follows.

(1) $$\begin{array}{c} {\text{EMG}}_{p}=\frac{{\text{EMG}}_{r}}{{\text{EMG}}_{\max}}, \end{array}$$

where EMG_*r*_ is real-time EMG signal displayed in real-time feedback bar and EMG_max⁡_ is the maximum EMG signal from MVC. EMG_*p*_ is proportional of EMG signals and displayed in real time to instruct the subjects.

A real-time feedback bar is shown to the subject for precise control of the EMG amplitude (as shown in Figure 2(b)). The subject should keep the activity of any other muscle than the target one as minimal as possible. For example, when ED is the target muscle, APB and FD should be in the rest state. In each trial, the durations of the ready state, action state, and rest state are 1 s, 40 s, and 5 s, respectively, (as shown in Figure 2(b)). The subject should be prepared in the ready state to reduce onset artifacts in the action state and minimize eye blinks and irrelevant movements during the action state. There are total 6 trials for each hand movements. The subjects should perform four hand movements: finger extension, thumb adduction, finger flexion, and rest.

### 2.2. EEG and EMG Acquisition

EEG signals are recorded from 64 scalp positions system using the international 10–20 system (Synamp2, Compumedics Inc., Charlotte, NC, USA) referenced to right mastoid and ground at AFz, and motor cortex related 35 positions are recorded (as shown in Figure 1(a)). EEG signals are filtered by a 1 Hz–200 Hz band-pass filter and sampling frequency is 1000 Hz. Before recording, the reference surface skin is prepared with neuroprep and alcohol to lower the impedance under 5 kΩ with Ag/AgCl electrodes.

EMG signals are recorded by surface electrodes with band-pass filter between 5 and 200 Hz and sampling frequency is 1000 Hz using neuroscan Synamp2 EMG acquisition. The Ag/AgCl electrodes are applied on three surface muscles (ED, APB, and FD) with using electrical stimulation which fixed the position. The distance between a pair of electrodes is 5 cm in ED and FD muscles, 1 cm in APB muscle. All electrode impedances are kept under 5 kΩ.

### 2.3. Signal Analysis

EOG is recorded in EEG and EMG acquisition at the same time to remove signals contaminated with eye movement. After removing artifacts and EOG in the EEG and EMG signals trial by trial, we partition the EEG and EMG signals into nonoverlapping segments of 1024 ms, which has a frequency resolution of 0.976 Hz. There are 150–190 seconds of available data collected for all subjects. Here we adopt uniformly 150 segments to estimate CMC value over the whole frequency range. Coherence spectrum is calculated with a fast Fourier transform algorithm:

(2) $$\begin{array}{c} C_{}\left(f,\text{ch}\right)=\frac{C_{xy}\left(f,\text{ch}\right)}{C_{xx}\left(f,\text{ch}\right)C_{yy}\left(f,\text{ch}\right)}, \end{array}$$

where *f* is frequency and ch is channel. *C*_*xy*_(*f*, ch) is cross-spectrum of EEG and EMG, and *C*_*xx*_(*f*, ch) and *C*_*yy*_(*f*, ch) are autospectrum of EEG and EMG signal, respectively. Therefore, CMC is function of frequency and channel. The confidence level is calculated in

(3) $$\begin{array}{c} {\text{CL}}_{\partial }=1-{\left(1-\partial \right)}^{1/\left(N-1\right)}, \end{array}$$

where *α* is confidence level, *N* is the number of segments. (*α* is 95% in our study and correspondence to *P* value is 0.05; *N* is 150 in our study.) CL represents the confidential limit. If the value is above CL, the coherence is considered to be significant.

 In order to classify finger extension from thumb adduction, finger flexion, and rest, we explore the spatial and temporal distribution of CMC on ED muscle and EMG signals during the above-mentioned hand movements, which refers to channel and frequency respectively. For simple expression, here we define a few abbreviations below.

 CMC_FE_max⁡_ = ED muscle CMC peak value in finger extension movement. “FE” represents finger extension.
 CMC_TA_max⁡_ = ED muscle CMC peak value in thumb adduction movement. “TA” represents thumb adduction.
 CMC_FF_max⁡_ = ED muscle CMC peak value in finger flexion movement. “FF” represents finger flexion.
 CMC_REST_max⁡_ = ED muscle CMC peak value in rest.
 
*F*_FE_max⁡_ = the frequency which reaches CMC_FE_max⁡_.
 Ch_FE_max⁡_ = the channel which provides CMC_FE_max⁡_.

We first compare the maximal CMC value of ED muscle (CMC_FE_max⁡_ versus CMC_TA_max⁡_, and CMC_FE_max⁡_ versus CMC_FF_max⁡_) across the all frequency and all EEG channels to see whether there is a significant difference across subjects in ED CMC peak value in finger extension other than thumb adduction or finger flexion. Next, we then generate the topographical distribution of CMC on the scalp, find out the most related cortical area, which means that the channel is fixed at Ch_FE_max⁡_ and observe the maximal CMC value of ED muscle across the frequency range. *t*-test is applied to check if there is significant difference across subjects between finger extension and the 3 other movement tasks. Then, we obtain the tuning frequency of finger extension (*F*_FE_max⁡_) at the most related EEG channel. *t*-test is again used to check whether the ED CMC value acquired at Ch_FE_max⁡_ and *F*_FE_max⁡_ has a significant difference across subjects in finger extension from the other two above mentioned movements and rest.

If we could successfully classify the significant coherence of finger extension from other movement tasks, it provides promise to detect voluntary hand opening (versus hand closing) during active therapy. Here we divide signals into 20–30 segments for each subject (as shown in Table 2). The length of the segment is chosen when there is CMC value appeared above the significant value. We calculate CMC value within beta frequency between the collected EMG signals and the most related motor cortical channel. Linear discriminate analysis is applied on the CMC vector (across beta frequency) to calculate the classification accuracy in distinguishing finger extension from thumb adduction, finger extension, and rest for each subject. 

## 3. Results 

We first compare the maximal CMC value of ED muscle (CMC_FE_max⁡_ versus CMC_TA_max⁡_ and CMC_FE_max⁡_ versus CMC_FF_max⁡_) across the all frequency, and all EEG channels. With *t*-test, there is no significant difference in finger extension from thumb adduction and finger flexion in channel, frequency and peak coherence value.

**Figure 3:**
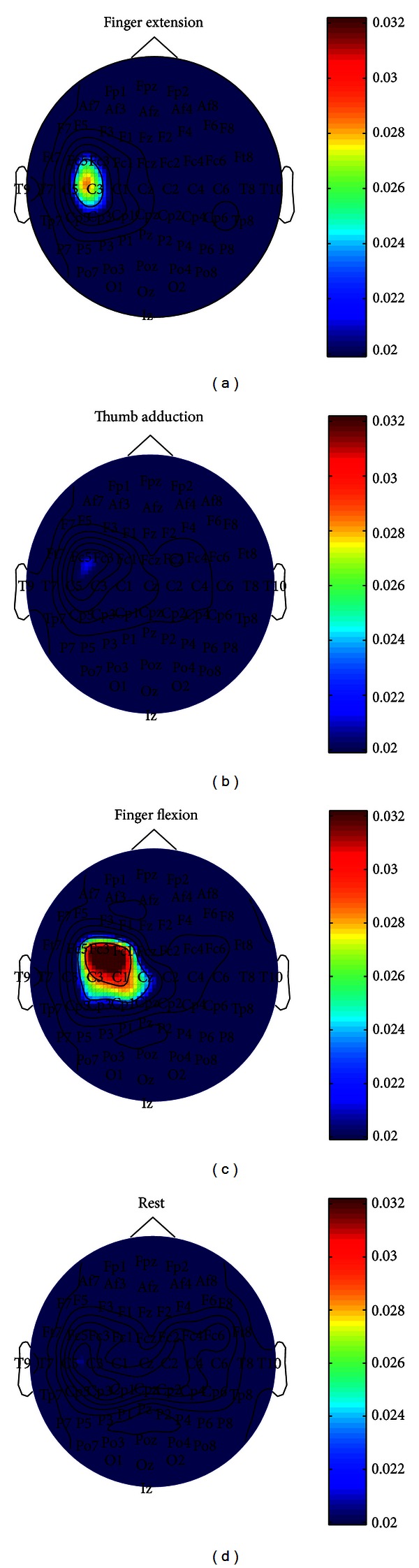
The topographical distribution of ED muscle CMC value on finger extension, thumb adduction, finger flexion, and rest. The significant ED muscle CMC has overlap in these mentioned movements around C3. The significant value was 0.0199, and the color bar was corresponding to CMC value bar. The peak coherence position and frequency are 15 and 20.51 Hz, respectively, in finger extension. The peak coherence position and frequency are 2 and 15.1367 Hz, respectively, in thumb adduction. The peak coherence position and frequency are 9 and 26.37 Hz, respectively, in finger flexion.

An example of the topographical distribution of ED CMC value in the different actions is shown in Figure 3. At *F*_FE_max⁡_, ED muscle CMC value is more obvious in finger extension and finger flexion than in thumb adduction, and rest. As seen from the Figure 3, the significant ED muscle CMC has overlap in channels on left motor cortex between finger extension with thumb adduction, finger flexion and rest.

We fix the channel at Ch_FE_max⁡_ and observe the CMC value across frequencies. We calculate the maximal ED muscle CMC value of four movement tasks across frequencies at Ch_FE_max⁡_ as shown in Table 3. With *t*-test, there is no significant difference between finger extension and finger flexion in ED CMC value. And there is no significant difference in finger extension from thumb adduction, finger flexion, and rest in frequency. But there is a significant difference at ED CMC value in finger extension from thumb adduction and rest; the *P* values are 0.0102 and 0.0161, respectively, at *α* = 0.05. 

**Figure 4:**
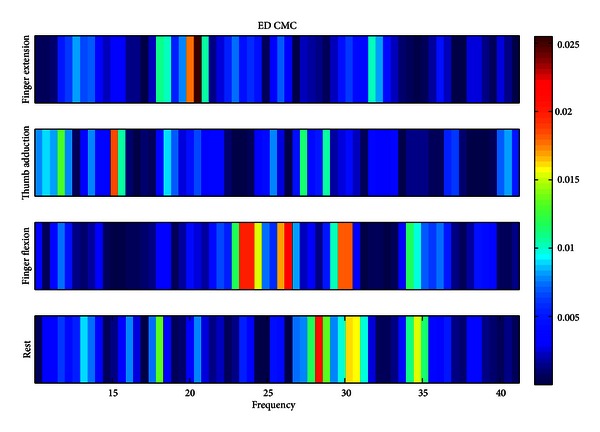
*Subject 3.* ED muscle CMC exists in finger extension, thumb adduction, finger flexion, and rest with peak appearing at different four hand movement tasks. ED muscle CMC peak value is in 20.51 Hz, 15.14 Hz, 26.37 Hz and 28.32 Hz for finger extension, thumb adduction, finger flexion, and rest, respectively.

Figure 4 shows an example of the distribution of ED CMC collected on Ch_FE_max⁡_ across beta frequency range for 4 different movement tasks. We can see that the ED CMC peak value appears at different frequencies for four movement tasks.

**Figure 5:**
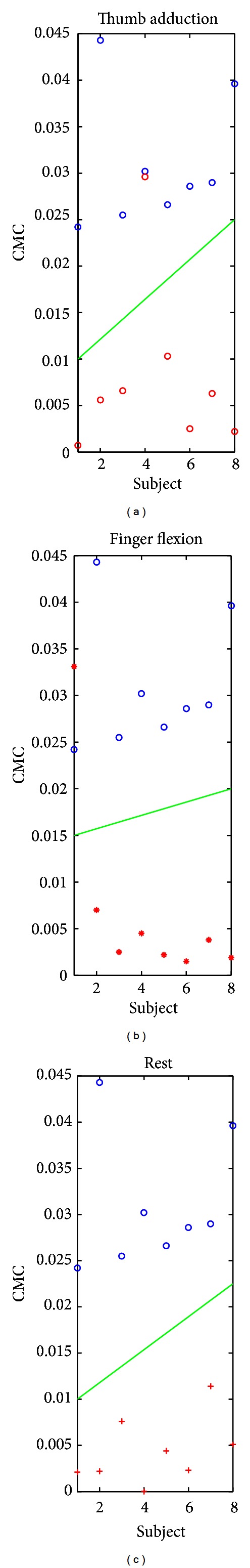
ED muscle CMC values at Ch_FE_max⁡_ and *F*_FE_max⁡_ in finger extension could be classified from thumb adduction, finger flexion, and rest. Finger extension versus thumb adduction (a). Finger extension versus finger flexion (b). Finger extension versus rest (c). Blue circle is ED muscle CMC values in finger extension. Red circle is ED muscle CMC value in thumb adduction. Red asterisk is ED muscle CMC value in finger flexion. Red cross is ED muscle CMC value in rest. Green l line distinctly classified ED EMC in finger extension from thumb adduction, finger flexion, and rest.

Then, we obtain the tuning frequency of finger extension (*F*_FE_max⁡_) at the most related EEG channel. The ED CMC values acquired at Ch_FE_max⁡_ and *F*_FE_max⁡_ for four movement tasks that are shown in Table 4. The result of *t*-test shows there is significant difference across subjects in finger extension from the other three movements in ED muscle CMC value with the *P* values are 0.000000205, 0.000089, and 0.00012 respectively at *α* = 0.05 (as shown in Table 4). It is obvious to linearly classify ED CMC in finger extension from other two movements and rest (as shown in Figure 5). 

**Figure 6:**
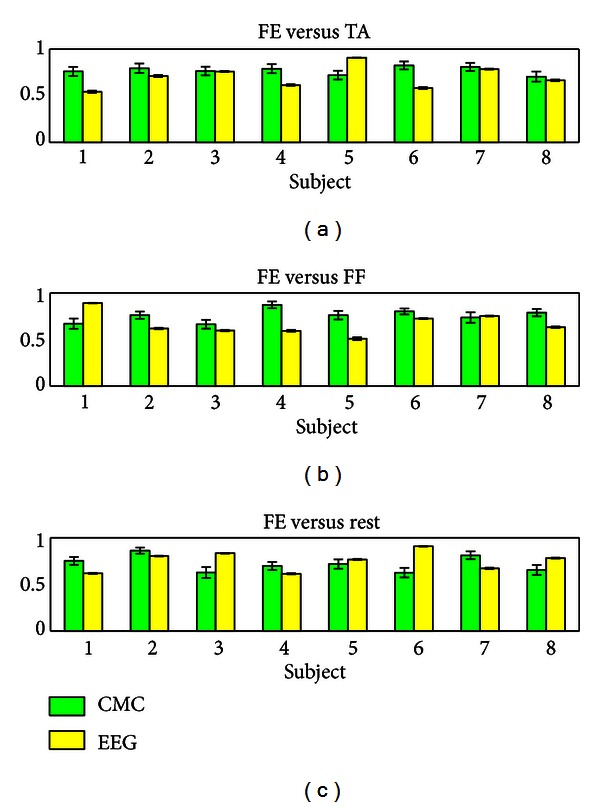
Classification accuracies are shown in the figure to distinguish finger extension from thumb adduction (a), finger flexion (b), and rest (c). FE, TA, FF are finger extension, thumb adduction and finger flexion, respectively. Green represents classification based on CMC and yellow represents classification based on EEG. The average accuracies by CMC and EEG are shown in Table 5.

Without too finely tuning on the frequency, we calculated CMC value within beta frequency between the collected EMG signals and the most related motor cortical channel. Linear discriminate analysis is applied on the CMC vector (across beta frequency) to classify finger extension from thumb adduction, finger extension, and rest for each subject. Fourfold cross-validation is adopted in LDA analysis. To show the superiority of our method, the classification results by CMC are compared with EEG in this study. This is because in healthy subject, finger extension could be easily classified from thumb adduction, finger extension and rest. But it is difficult in stroke patient due to abnormal coactive muscle and spasticity [22]. Study has shown that the average classification accuracy was 71.6% in moderately impaired subjects and only 37.9% in severely impaired subjects [22]. The voluntary intention extract from such signals may not be reliable. Therefore, good classification results by EMG do not necessarily result in good performance in patients. Here we classify finger extension from thumb adduction, finger flexion, and rest using CMC and by EEG only at the same channels (as shown in Figure 6).

The average accuracies across subjects by CMC are 78.96  ±  4.29%, 81.00 ± 7.34%, and 78.025 ± 9.39%, respectively, to distinguish finger extension from thumb adduction, finger flexion, and rest (see Table 5). It indicates the possibility to detect voluntary hand opening (versus hand closing) based on coherence analysis between EMG signal on one single muscle and one EEG channel located on motor cortical area. The average accuracies by EEG across subjects are 71.27 ± 12.32%, 71.12 ± 12.80%, and 81.39 ± 11.52%, respectively, (see Table 5). CMC classification accuracy is around 10% higher in average and less variance than EEG classification in thumb adduction and finger flexion, which is the main function (hand opening) we focused on for hand rehabilitation. The classification results on finger extension versus rest by CMC and EEG are similar (less average and less variance), which matches the good performance by EEG to distinguish movement from rest [39].

Furthermore, we do the 3-class classification (finger extension versus thumb adduction versus finger flexion) to extract finger extension. The best two subjects' accuracies by CMC are 75.80% and 79.56%.  The mean accuracy of the 8 patients by CMC is 69%, which is greater than 64% by EEG.

## 4. Discussion and Conclusion

Active rehabilitation involves patients' voluntary movement intention. It is effective to restore stroke patient' motor function, such as hand opening (one of the most challenge movements) [23, 24]. In this study, we focus on finger extension, which is mainly involved in hand opening. EMG could be easily to extract such intention, while it is not reliable to extract such intention due to the abnormal coactive muscles and spasticity in stroke patient [22]. EEG can be used to interpret patient movement intentions, but it is not good enough to distinguish ipsilateral hand movements [28–31]. Cortico-muscular coherence exists between EEG and EMG even on the abnormal muscle when stroke patient's intention appears [40]. We propose to analyze cortico-muscular coherence between EMG and EEG signals to distinguish voluntary ipsilateral hand opening, hand closing, and rest state. The EMG signals of Extensor Digitorum (ED) muscle and EEG signals are simultaneously collected when 8 subjects are instructed to voluntarily execute right finger extension, thumb adduction and finger flexion, and rest. We observe significant cortico-muscular coherence appearing at the left motor cortical area of the EEG channels, which is consistent with findings in Mima and Hallett's study [36] and shows different patterns within beta frequency range for 4 different executions. Statistical *t*-test shows that the coherence values of finger extension collected at tuning frequency and the most related channel are statically different from 3 other states, respectively, across all subjects. We apply linear discriminate analysis on the coherence pattern within beta range and average accuracy to distinguish finger extension state from thumb adduction, finger flexion, and rest. CMC classification accuracy is around 10% higher in average and less variance than the performance using EEG only in distinguishing finger extension from thumb adduction and finger flexion, which is the main function (hand opening) we focused on for hand rehabilitation. The classification results on finger extension versus rest by CMC and EEG are similar (less average but less variance), which matches the results that EEG can be used to distinguish movement from rest [39]. Furthermore, the classification results on finger extension out of 3 classes (finger extension versus thumb adduction versus finger flexion) by CMC are also greater than the performance by EEG only.

The results indicate the possibility to detect voluntary hand opening based on coherence analysis between one single muscle EMG signal and one EEG channel located on motor cortical area. One of the challenges is to accurately capture the instantaneous CMC value in real-time recoding. In the real application, EEG and EMC signals can be recorded from the patients at the same time. The significant coherence (CMC) value can be estimated to better classify the voluntary finger extension than EEG only and used as a more accurate control signal to evoke the electrical stimulation in active rehabilitation for hand movement. With further experiments on stroke patients, CMC needs to be compared with EMG on abnormal muscles to classify the hand movement. It will eventually help develop a new rehabilitation protocol that can benefit the hand rehabilitation for stroke survivors.

## Figures and Tables

**Table 1:** Character of subjects.

Subject	Age	Gender	Handedness
1	25	Male	Right
2	27	Male	Right
3	24	Male	Right
4	24	Male	Right
5	23	Female	Right
6	23	Male	Right
7	24	Male	Right
8	23	Male	Right

**Table 2:** Number of segments for each subject.

Subject	1	2	3	4	5	8	7	8
Number of segments	25	23	25	22	20	25	21	23

**Table 3:** ED muscle CMC on four movements at Ch_FE_max_ (Ch is channel).

Subject	Ch	Finger extension		Thumb adduction		Finger flexion		Rest	
		Peak	Frequency	Peak*	Frequency	Peak	Frequency	Peak*	Frequency
		CMC	(Hz)	CMC	(Hz)	CMC	(Hz)	CMC	(Hz)
1	24	0.0242	35.64	0.0234	35.64	0.0377	35.16	0.0185	17.09
2	9	0.0443	15.63	0.0207	37.10	0.0356	30.27	0.0192	41.02
3	15	0.0255	20.51	0.0187	15.13	0.0222	26.37	0.0204	28.32
4	2	0.0302	12.70	0.0296	12.70	0.0346	10.25	0.0300	33.20
5	9	0.0266	36.62	0.0221	33.69	0.0128	12.70	0.0248	29.97
6	24	0.0286	13.18	0.0243	10.25	0.019	25.88	0.0255	30.76
7	16	0.029	37.60	0.0172	13.18	0.0116	32.23	0.0207	40.53
8	24	0.0396	21.00	0.024	13.67	0.0273	31.25	0.0211	10.25
Mean	/	0.031	24.11	0.0225	21.42	0.0251	25.51	0.0225	29.89
SD	/	0.0071	10.80	0.0038	11.75	0.0103	9.19	0.0039	10.64

*Represents that there is significant difference compared with finger extension. The ED CMC value compared in finger extension from thumb adduction and rest is significant and the *P* values are 0.0102 and 0.0161, respectively, at *α* = 0.05.

**Table 4:** ED muscle CMC value in four movements at Ch_FE_max_ and F_FE_max_.

Subject	Channel	Frequency (Hz)	Finger extension	Thumb adduction	Finger flexion	Rest
			Peak*	Peak*	Peak*	Peak*
1	24	35.64	0.0242	0.000736	0.0331	0.0021
2	9	15.63	0.0443	0.0056	0.007	0.0022
3	15	20.51	0.0255	0.0066	0.0025	0.0076
4	2	12.70	0.0302	0.0296	0.0045	0.000017
5	9	36.62	0.0266	0.0103	0.0022	0.0044
6	24	13.18	0.0286	0.0025	0.0015	0.0023
7	16	37.60	0.029	0.0063	0.0038	0.0114
8	24	21.00	0.0396	0.0022	0.0019	0.0051
Mean	/	24.11	0.0310	0.0080	0.0071	0.0044
SD	/	10.79	0.0071	0.0093	0.0107	0.0037

*represents that there is significant difference compared with finger extension. The *P* values are 0.000000205, 0.000089, and 0.00012, respectively, at *α* = 0.05.

**Table 5:** Average accuracies of CMC and EEG classification.

	FE versus TA	FE versus FF	FE versus REST
CMC	78.96 ± 4.29%	81.00 ± 7.34%	78.025 ± 9.39%
EEG	71.27± 12.32%	71.12 ± 12.80%	81.39 ± 11.52%
